# Neural correlates of food labels on brand, nature, and nutrition: An fMRI meta-analysis

**DOI:** 10.3389/fnut.2022.1056692

**Published:** 2022-12-20

**Authors:** Andy Wai Kan Yeung

**Affiliations:** Oral and Maxillofacial Radiology, Applied Oral Sciences and Community Dental Care, Faculty of Dentistry, The University of Hong Kong, Hong Kong, Hong Kong SAR, China

**Keywords:** food, nutrition, meta-analysis, gustation, taste, CBMA food, CBMA

## Abstract

Eating is an essential act of our everyday life, and it involves complicated cognitive appraisal and gustatory evaluation. This study meta-analyzed the functional magnetic resonance imaging (fMRI) studies about food labels on brand, nature and nutrition. Web of Science Core Collection (WoS), Scopus, and PubMed were queried to identify human fMRI studies written in English and published in peer-reviewed journals and used taste or food related labels. Studies were excluded if they reported no results from taste/food related stimuli versus control, no task-based fMRI results, or no results from whole-brain analysis. Nineteen studies entered the analysis. Results for the meta-analysis on food nutrition revealed that the precuneus on the right hemisphere was significantly activated, a brain region related to internal mentation of self-consciousness and nutritional evaluation. Results for the overall analysis on all 19 studies, the analysis on food brand, and the analysis on food nature revealed no significant brain regions. Food nutrition labels were generally processed by brain regions related to internal mentation of self-consciousness and nutritional evaluation. However, the neural correlates of labels of food brand and food nature were inconsistent across studies. More future studies are needed to better understand the cognitive processing of different kinds of food labels in our brain.

## Introduction

Eating is an essential act of our everyday life, and it involves complicated cognitive appraisal and gustatory evaluation. Multiple meta-analyses have attempted to pool data across studies to identify brain regions responsible for primary tasting (1–3); for processing visual, taste, and odor food stimuli (4), food commercials (5), affect, intensity, and quality of food stimuli (6), tastants delivered with and without swallowing (7); for differential processing between hungry and satiated state (8), male and female (9), obese and normal weight (10); and regions targeted by anti-appetite medications (11). (For a more complete list of meta-analyses on taste and food, please refer to (12)). Meanwhile, different kinds of food label, such as food brand logo, nutritional label, or label about food nature should modulate the cerebral processing of food information. An obvious analogy is graphic cigarette warning label. Some smokers and potential smokers may refrain from taking the cigarettes upon seeing such warning label printed on a pack of cigarettes. Compared to control warnings, it was reported that such graphic warning labels could significantly improve subjects’ motivation to quit smoking, and elicited stronger activation in numerous brain regions (13). The information provided by a nutritional label may similarly influence consumer behavior. For example, consumers were likely to pay 11% more for a box of cookies with a nutritional label compared to its counterpart without a label (14). There could be more modulating factors such as consumers’ nutritional knowledge and trust toward the nutrition and health claims (15, 16). On the other hand, food brand could also affect how much a consumer liked the product and his/her subsequent purchasing behavior (17). Understanding the neurobiology underlying such food choice and consumption behavior is obviously beneficial for marketing, nutritional advice, and pharmaceuticals. For instance, the cerebral regions highly related to gustatory processing, such as the insula and orbitofrontal cortex, were activated in response to visual cues of shower gel and dish liquid with beverage-imitating packages (18). This might explain why some people would unintentionally drink shampoo or other poisonous home products. In fact, anti-appetite medications often targeted the gustatory processing at the insula (11). Besides, it was found that discrete regions of the prefrontal cortex, responsible for decision making and self-control, were activated in response to seeing popular brand logos of foods such as French fries versus corresponding organic brand logos (19). It is important to know more about the neural correlates of various types of food labels, so that the designs of the food labels can be tested and optimized by neuroimaging evaluations.

Here, three types of food labels were examined: food brand logo, nutritional label, or label about food nature. Food brand logos might trigger a consumer’s memory about the past consumption experience and involve social facets such as brand loyalty. In 1985, the Coca Cola company introduced the so-called “New Coke” into the market, which was rated more superiorly than the original product in blinded taste tests. However, the word “new” printed on the can was largely rejected by consumers as they preferred the original image of the can and thus the original “package” of Coca Cola including the drink filled into the can (20). Similarly, it was found that human brain had differential gustatory processing of Coca Cola relative to Pepsi in multiple regions such as dorsolateral prefrontal cortex and hippocampus, implying differential drink preference (21). Meanwhile, nutritional label provides nutritional information of the food product that may influence liking, perception of healthiness, and ultimately food choice. There are three main groups of nutritional labels, traffic light, guideline daily amounts (GDA), and health logos/ratings (22). Consumers were more willing to pay for foods tagged with healthy nutritional labels than foods with unhealthy labels (23). In particular, seeing foods tagged with red (unhealthy) traffic light nutritional label would activate the inferior frontal gyrus and dorsolateral prefrontal cortex, regions implicated in self-control in food choice (23). Finally, the remaining type of label is about food nature. Food nature has diverse context. One fundamental aspect is food versus non-food. For instance, isovaleric acid is a chemical with a strong odor. It is present in foods (e.g., cheese and apple juice) and also produced by human body, attributing to sweaty smell and foot odor (24). The smell of isovaleric acid was rated much more unpleasant when it was labeled as body odor compared to labeled as cheddar cheese, with differential activations at the anterior cingulate, medial orbitofrontal cortex, and the amygdala (25). Apart from food versus non-food, the social/environment meanings of the food can also be expressed by the labels, such as Halal versus non-Halal for Muslims (26) was differentiated in the ventromedial prefrontal cortex, organic versus conventional (27) was differentiated in the ventral striatum, and fair trade versus conventional (28) was differentiated in the ventral striatum, cingulate cortex, and superior frontal gyrus.

Therefore, this meta-analytic study pooled data across functional magnetic resonance imaging (fMRI) studies. The activation likelihood estimation (ALE) meta-analytic approach was selected, which calculated the consistency of significantly activated brain coordinates reported from published studies by means of probability distribution modeling (29, 30). Based on the existing literature, it was hypothesized that the neural correlates of food brand logo, nutritional label, and label about food nature would involve the gustatory network, such as the insula and the prefrontal cortex.

## Materials and methods

### Literature search and study selection

**FIGURE 1:**
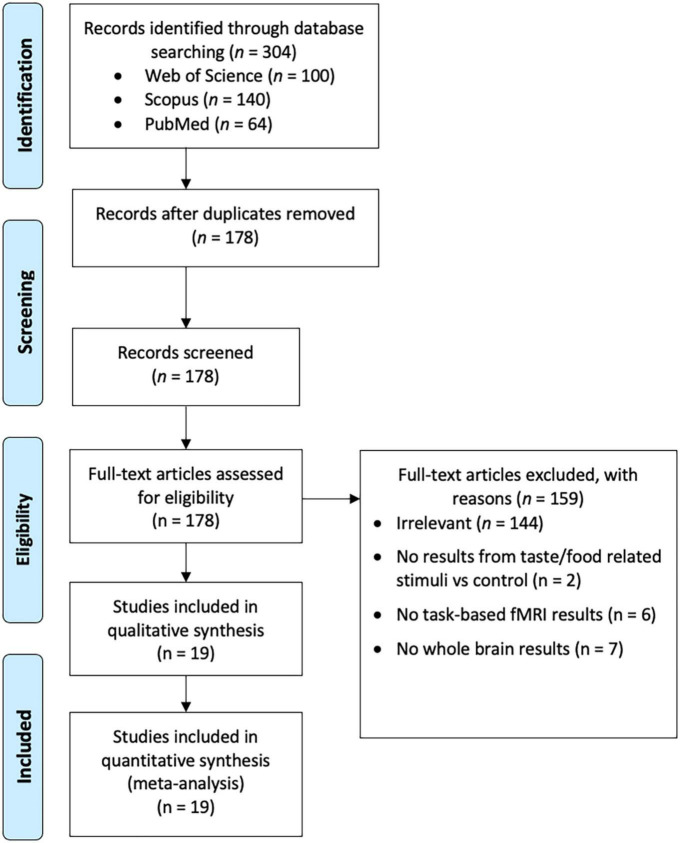
A flow chart that illustrates the literature search.

In May 2022, three databases, Web of Science Core Collection (WoS), Scopus, and PubMed were queried with the following search string: (taste* OR flavo* OR food*) AND (fMRI OR neuroimaging OR “functional MRI” OR “functional magnetic”) AND (brand* OR label* OR logo*). For the former two databases, the title, abstract, and keyword fields were searched. Since PubMed does not index keywords, the title and abstract fields were searched. The search yielded 100 papers from WoS, 149 from Scopus, and 64 from PubMed. After excluding duplicates, 178 papers remained. The titles and abstracts of these 178 papers were reviewed to determine their relevance. Papers were eligible if they were (i) original studies written in English and published in peer-reviewed journals; (ii) human fMRI studies; and (iii) using taste or food related labels. Studies were excluded if they reported (i) no results from taste/food related stimuli versus control; (ii) no task-based fMRI results; or (iii) no results from whole-brain analysis. Finally, 19 studies remained (19, 21, 23, 25, 27, 28, 31–43) (Figure 1 and Table 1). This study was not pre-registered in PROSPERO or other databases.

**TABLE 1:** Details of the 19 analyzed studies.

References	Journal	Sample size	Handedness	Mean age ± SD (range)	Mean BMI ± SD (range)	Hours fasted	Dimensions investigated	Test stimuli	Control stimuli	Contrast with significant results	Foci	Data location in the original paper
(21)	Neuron	16 (?M ?F)	?	28.0 ± 7.6 (19–50)	?	?	Food brand	Coca Cola image + Coca Cola tasting	Colored circle + Coca Cola tasting	Test > Control	7	Table 1
(25)	Neuron	12 (12M)	R	(23–35)	?	?	Food nature	Words of “cheddar cheese” + odor (isovaleric acid combined with cheddar cheese flavor)	Words of “body odor” + odor (isovaleric acid combined with cheddar cheese flavor)	Test > Control	3	P673
(27)	NeuroImage	30 (15M 15F)	R	26.0 (23–38)	?	4	Food nature	Food images with organic food emblem	Food images with conventional food emblem	Test > Control	2	P217
(31)	Brain Res	12 (7M 5F)	R	25 (21–31)	?	?	Food brand	Chocolate images from brands attractive to subjects	Chocolate images from non-attractive brands	Test > Control	2	P84
(32)	PLOS One	15 (8M 7F)	?	31.4 (23–50)	?	?	Food brand	Images + tasting of famous brands Cola	Images + tasting of unfamous brands Cola	Test > Control	1	3rd page
(33)	Soc Cogn Affect Neurosci	17 (10M 7F)	?	11.8 ± 1.4 (10–14)	?	4	Food brand/nature	Images of food brand logos	Images of non-food brand logos	Test > Control	4	Table 2
(34)	Obesity	25 (13M 12F)	24R 1L	15.2 ± 0.8	22.8 ± 4.4	4	Food brand	Images of Coca Cola product and logo	Images of non-food images	Test > Control	19	Table 4^a^
(23)	Obesity	25 (11M 14F)	?	23.3 ± 4.4 (19–37)	23.1 ± 3.4 (17–33)	Did not fast	Food nutrition	Food images with healthy nutritional labels	Food images with unhealthy nutritional labels	Both ways	9	Supplementary Table 1^b^
(28)	Front Behav Neurosci	33 (?M ?F)	Mostly R	24.1 ± 3.6 (19–33)	?	?	Food nature	Food images with Fair Trade emblem	Food images with no emblem	Test > Control	9	Table 1
(35)	J Culin Sci Technol	9 (9M)	R	26 ± 9 (18–45)	?	?	Food nature	Images of unfamiliar food with name label	Images of unfamiliar food with no label	Both ways	10	Table 2^b^
(36)	Am J Clin Nutr	20 (10M 10F)	?	23.3 ± 3.4	22.1 ± 1.9	?	Food brand	Images of familiarized food brand logo	Images of unfamiliar food brand logo	Test > Control	3	Table 1
(19)	Cogent Psychol	23 (14M 9F)	R	37.1 ± 7.6 (27–47)	23.6 ± 1.7	2	Food brand/nature	Images of conventional food brand logo	Images of organic food brand logo	Both ways	4	Tables 3, 4^b^
(37)	PLoS One	42 (9M 33F)	Mostly R	19.6 ± 1.4 (18–22)	23.4 ± 3.4	?	Food nutrition	Food images with calorie content label	Food images with no label	Both ways	11	Table 3^b^
(38)	Nutr Neurosci	50 (23M 27F)	48R 2L	26.5 ± 4.9	?	?	Food nutrition	Food images with 5 pieces of nutritional information displayed	Food images with 1 piece of nutritional information displayed	Test > Control	12	Tables 3, 4^a^
(39)	NeuroImage	31 (15M 16F)	R	24 (20–32)	23.1 (20.3–28.1)	6	Food nutrition	Words of “high calorie”	Words of “low calorie”	Test > Control	4	Table 3^a^
(40)	Neuropsychologia	40 (20M 20F)	R	20.7 ± 2.3 (18–27)	?	?	Food nature	Potato chip packaging image with a wrong match between flavor type and color	Potato chip packaging image with a correct match between flavor type and color	Test > Control	3	Table 2^a^
(41)	Brain Imaging Behav	25 (12M 13F)	R	8.6 ± 1.1 (7–10)	17.7 ± 2.8	2	Food brand/nature	Images of food brand logos	Images of non-food brand logos	Test > Control	2	Tables 4, 5
(42)	Nutrients	44 (44F)	R	20.0 ± 1.5	?	?	Food nutrition	Unhealthy food images with “traffic light” nutrition label	Unhealthy food images with “guideline-daily amount” nutrition label	Test > Control	6	Table 1
(43)	Cogn Affect Behav Neurosci	28 (15M 13F)	R	21.2 ± 2.1	21.7 ± 3.6	1	Food nature	Unhealthy food images + words of “eating together”	Unhealthy food images + words of “eating alone”	Test > Control	2	7th page

^a^Foci from 2 contrasts merged into 1 for the meta-analysis.

^b^Two contrasts extracted for the meta-analysis.

### ALE meta-analysis

**FIGURE 2:**
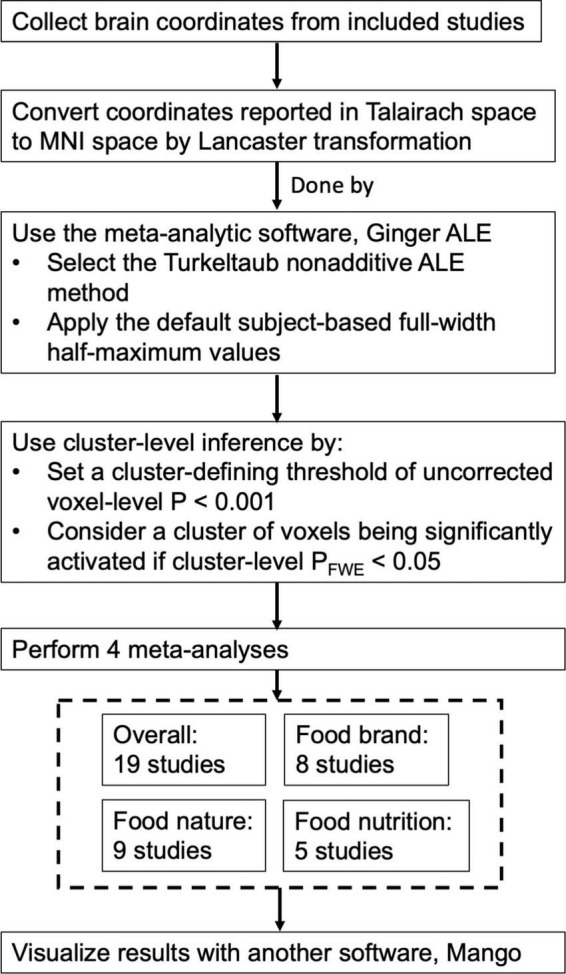
Flow chart that illustrates the process of activation likelihood estimation (ALE) meta-analysis.

Brain coordinates of reported activations were collected from the included studies. They were subjected to probability distribution modeling to unveil brain regions consistently activated across studies (29). Some studies mentioned that their coordinates were reported in Talairach space, while other studies reported their coordinates in Montreal Neurological Institute (MNI) space. For those coordinates reported in Talairach space, the Lancaster transformation (44) was applied to convert them into MNI space before the analysis. The software GingerALE (version 3.0.2) (45) was used for such conversion and to conduct the activation likelihood estimation (ALE) meta-analysis. The Turkeltaub non-additive ALE method was selected (46), and subject-based full-width half-maximum values were applied as default (29). For statistical threshold, a cluster-level inference was used. In other words, a cluster of voxels was considered significantly activated across the studies if it survived a cluster-level *P* < 0.05, familywise error (FWE) rate-corrected for multiple comparisons, with a cluster-defining threshold of uncorrected voxel-level *P* < 0.001. This is the standard statistical threshold recommended for ALE meta-analysis (47). A meta-analysis was performed independently for: (1) all 19 included studies, (2) 8 studies that involved food brand, (3) 9 studies that involved food nature, and (4) 5 studies that involved food nutrition. The thresholded ALE maps were overlaid onto the standard Colin T1 brain template in MNI space (48) and visualized with Mango 4.0 (49). The cluster size of brain regions was outputted by GingerALE from the ALE map. Figure 2 shows a flow chat that illustrated the process of this ALE meta-analysis.

### Label-based meta-analysis

In addition, a “label-based meta-analysis” [method based on (50)] was performed to tabulate/summarize the activated brain regions commonly reported across the studies. The extracted activation foci were re-surveyed by entering them into Mango 4.0, which provided a label to each focus (coordinate) to the nearest gray matter in a standardized way (50). This qualitative analysis would provide additional information to the readers in case the quantitative ALE analysis yielded insignificant results.

## Results

The details of the 19 fMRI studies on food labels are listed in Table 1. Each study recruited 9–50 subjects, with two studies recruiting men only and one study women only. Regarding subject handedness, 10 studies recruited right-handed subjects only, whereas 4 studies recruited mostly right-handed subjects and 5 studies did not report details. Regarding subject age, most of the studies recruited young and middle-aged adults, whereas 3 studies focused on children and adolescents below the age of 18, and no study involved the elderly above the age of 65. For body mass index (BMI) of the subjects, 8 studies provided the mean data that ranged from 17.7 to 23.6. Meanwhile, 7 studies reported the number of hours the subjects fasted prior to the fMRI scan (1–6 h) and 1 study reported that the subjects did not fast. For the dimensions investigated, 5 studies investigated food brand, 6 studies investigated food nature, 5 studies investigated food nutrition, and 3 studies investigated food brand/nature.

For ALE meta-analysis, the overall analysis on all 19 studies, the analysis on food brand, and the analysis on food nature revealed no significant brain regions. Results remained insignificant even when a more liberal threshold of *P* < 0.05 (false-discovery rate corrected) with cluster size > 200 mm^3^ was applied.

**FIGURE 3:**
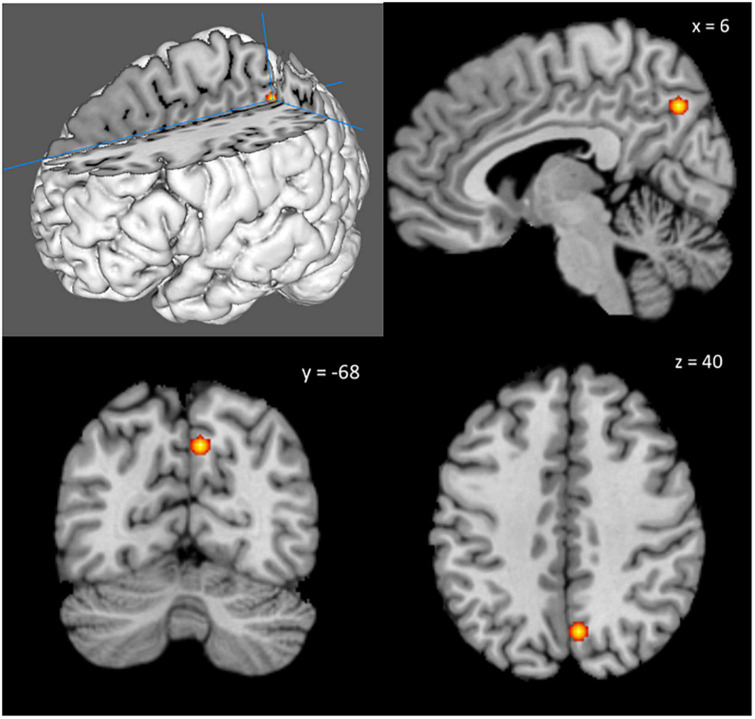
The right precuneus was significantly activated across studies that investigated food nutrition labels. The peak voxel of the activated cluster was at [6, –68, 40], with ALE value of 1.75 × 10^–2^ and cluster size of 520 mm^3^.

The ALE meta-analysis on food nutrition revealed that the precuneus on the right hemisphere was significantly activated (Figure 3). The peak voxel of the activated cluster was at [6, −68, 40], with ALE value of 1.75 × 10^–2^ and cluster size of 520 mm^3^.

**FIGURE 4:**
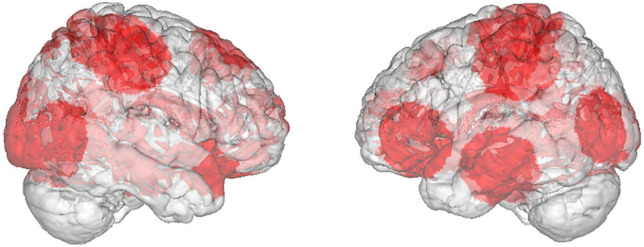
Unthresholded ALE brain map resulted from food brand studies showing the diverse brain regions involved. It is overlaid on a glass brain for visualization. None of the clusters reached statistical significance.

The additional label-based meta-analysis showed that the activated brain regions for food brand processing seemed to be more diverse/inconsistent compared to food nature and food nutrition (Tables 2–4). Figure 4 shows the unthresholded ALE brain map for food brand. It illustrated that multiple brain regions were reported from the included studies, though none of them reached statistical significance.

**TABLE 2:** Qualitative label-based meta-analysis of the eight studies concerning food brand.

	Parahippocampus	Substantia nigra	Superior frontal G	Thalamus	Anterior cingulate	Posterior cingulate	Lentiform nucleus	Caudate	Middle occipital G	Lingual G	Paracentral lobule	Postcentral G	Declive	Fusiform G	Precuneus	Middle temporal G	Inferior parietal lobule	Claustrum	Medial frontal G	Inferior frontal G
(21)	B	L	R	R		L														
(31)					R		R													
(32)								L												
(33)									R	L	R	L								
(34)							L		L	L		R	B	B	R	R	B	L		
(36)																L			R	
(19)					R							L								B
(41)														R						R

G, gyrus; B, both sides; R, right side; L, left side.

**TABLE 3:** Qualitative label-based meta-analysis of the nine studies concerning food nature.

	Middle occipital G	Inferior occipital G	Lingual G	Fusiform G	Paracentral lobule	Postcentral G	Precentral G	Lentiform nucleus	Middle frontal G	Medial frontal G	Inferior frontal G	Anterior cingulate	Cuneus	Culmen	Insula	Caudate	Claustrum	Transverse temporal G
(25)										R	R	L						
(27)								L	R									
(33)	R		L		R	L												
(28)			B						L		L	R	R		L	L		
(35)		R					L		B	R			L	L	R			
(19)						L					B	R						
(40)			L			R	R											
(41)				R							R							
(43)																	L	R

G, gyrus; B, both sides; R, right side; L, left side.

**TABLE 4:** Qualitative label-based meta-analysis of the five studies concerning food nutrition.

	Middle occipital G	Inferior occipital G	Lingual G	Fusiform G	Superior parietal lobule	Inferior parietal lobule	Postcentral G	Precentral G	Lentiform nucleus	Superior frontal G	Middle frontal G	Medial frontal G	Inferior frontal G	Anterior cingulate	Precuneus	Cuneus	Fusiform gyrus	Culmen	Insula	Declive	Middle temporal G	Superior temporal G
(23)		L				R				R	L		R	L	R	R						
(37)	L		L			B	R	R					R			R			R		R	
(38)	R	L			R					R	R		R		L		R			L		
(39)																					R	B
(42)	R		R							R		L		L						R		

G, gyrus; B, both sides; R, right side; L, left side.

## Discussion

This is the first meta-analysis on fMRI studies regarding brain responses to food labels. Surprisingly, results did not yield significant activations in the hypothesized brain regions of the insula and the prefrontal cortex. Results have shown that the cerebral processing of food labels on nutrition significantly activated the right precuneus. The processing of food labels on food brand and food nature did not yield significant results.

The insula is known to be the primary taste cortex that processes neural signals relayed from taste receptors in the oral cavity (51). The meta-analytic results here did not find significant activation of it, which might be partly explained by the fact that the analyzed studies did not involve actual tasting or stimulation of the taste receptors. Instead, the stimuli involved visual and cognitive aspects of food processing. Meanwhile, the prefrontal cortex is responsible for top-down affective modulation (51) and should be activated. The insignificant results might be attributed to heterogeneous designs of the studies.

The precuneus was reported to consume much more glucose than other parts of the cerebral cortex and thus sensitive to and easily affected by malnutrition (52). Among healthy subjects, precuneus was found to activate more in response to personalized nutritional messages compared to untailored nutritional messages (53). Meanwhile, its cortical thickness correlated with nutritional state and cognitive functions in patients with anorexia nervosa (54). Hence, the results of current meta-analysis on food nutrition labels were consistent to the existing literature that the precuneus seems to play an important role in nutritional evaluation.

At first glance, the insignificant result from food brand was unexpected. However, a prior fMRI study that presented visual cues of car brands showed that the cerebral processing of brands indeed involved diverse brain regions, e.g., familiar brands preferentially activated superior frontal gyrus, hippocampus and posterior cingulate; sports and luxury brands activated medial prefrontal cortex and precuneus; whereas brands deemed value products activated superior frontal gyrus and anterior cingulate (55). Therefore, the heterogeneity of subject background and the exact instructions given to them during fMRI scan might have attributed to the inconsistency across the analyzed studies. As a matter of fact, one of the analyzed studies on food brand did investigated Coca Cola and Pepsi separately, and compared brand-cued drink delivery to light-cued (colored circle) delivery (21). Significant results were only found for Coca Cola but not Pepsi. Another study similarly found that Coca Cola elicited greater activation than Pepsi in the right amygdala, whereas the opposite comparison resulted in no significant activation (32). This illustrated that the cerebral processing of food brand(s) could be complicated and not easily elicited in a consistent manner.

The heterogeneous situation was more obvious for food nature studies. Three studies investigated food versus non-food labels, followed by two investigated organic versus conventional food. Other comparisons were each investigated in one study, such as fair-trade label versus no label, food name label versus no label, eating alone label versus eating together label, and a correct match between flavor name and color on potato chip packaging versus a wrong match. It could be reasonably argued that different brain regions could be utilized for the cognitive processing of such distinct group of labels about food nature, rendering the results insignificant. There were even other labels on food nature being investigated. For instance, it was reported that there was a significant difference in the ventromedial prefrontal cortex activity level between viewing meat images labeled with Halal logo and those labeled with non-Halal logo, though this study was not included in the present analysis due to its lack of description on whether whole-brain analysis was performed (and also no brain coordinates provided) (26).

This meta-analysis has several limitations. First, the participants involved in the analyzed studies were mainly young and middle-aged adults with some children and adolescents. The results therefore might not be well applied to the elderly population, especially that aging was shown to associate with decreasing activation in the precuneus in response to food stimuli in a previous study (56). In addition, the analyses on the food brand and food nature subsets did not reveal significant results, so that potential follow-up analyses to compare between food brand, food nature, and food nutrition could not be conducted. Moreover, the majority of the analyzed studies exclusively recruited right-handed subjects or did not report subject handedness, similar to the scenario revealed by a prior survey on nutritional neuroscience literature (57). It was established that handedness would affect the lateralization of cerebral processing of tasting (58, 59). However, the methodology to gauge and determine subject handedness lacked a consensus and remained disputable (60). Regardless, due to the lack of results from exclusively left-handed subjects, it was not possible to test and determine if handedness would affect cerebral processing of food labels. Readers should be aware of these limitations during results interpretation. Finally, ALE meta-analysis can only compute the consistency of brain coordinates activated across studies but it cannot compute the effect size (30). Subsequently, future studies should include left-handed subjects in their sample. Moreover, the elderly people should be recruited so that the results could be more representative of the whole population. Researchers should share the statistical brain maps resulted from future original studies or report their effect size, so that the effect size could also be meta-analyzed.

In conclusion, the cerebral processing of food labels on nutrition significantly activated the right precuneus, a brain region related to nutritional evaluation. The peak voxel of the activated cluster was at [6, −68, 40], with ALE value of 1.75 × 10^–2^ and cluster size of 520mm^3^. The processing of food labels on food brand and food nature did not yield significant results. More future studies are needed to better understand the cognitive processing of different kinds of food labels in our brain. They should include left-handed subjects as well as the elderly people, so that results could be more generalizable.

## Data availability statement

The original contributions presented in this study are included in the article/supplementary material, further inquiries can be directed to the corresponding author.

## Author contributions

The author confirms being the sole contributor of this work and has approved it for publication.
